# Comparative Effects of Dulaglutide and Semaglutide on Renal Function Decline and Proteinuria Reduction in Diabetic Patients: A Retrospective Cohort Study

**DOI:** 10.3390/jcm14124287

**Published:** 2025-06-16

**Authors:** Yuh-Mou Sue, De-En Lu, Te-I Chang, Chun-You Chen, Cheng-Hsien Chen, Shih-Chang Hsu, Yen-Ling Chu, Nai-Jen Huang, Tso-Hsiao Chen, Feng-Yen Lin, Chun-Ming Shih, Po-Hsun Huang, Hui-Ling Hsieh, Chung-Te Liu

**Affiliations:** 1 Department of Internal Medicine, School of Medicine, College of Medicine, Taipei Medical University, Taipei 110, Taiwan; sueym@tmu.edu.tw (Y.-M.S.); hippy@tmu.edu.tw (C.-H.C.); 88128@w.tmu.edu.tw (T.-H.C.); g870905@tmu.edu.tw (F.-Y.L.); cmshih53@tmu.edu.tw (C.-M.S.); 2 Division of Nephrology, Department of Internal Medicine, Wan Fang Hospital, Taipei Medical University, Taipei 116, Taiwan; 108100@w.tmu.edu.tw (D.-E.L.); e516091013@tmu.edu.tw (Y.-L.C.); m118108006@tmu.edu.tw (N.-J.H.); 3 Division of Nephrology, Hsin Kuo Min Hospital, Taipei Medical University, Taoyuan 320, Taiwan; 4 Department of Surgery, School of Medicine, College of Medicine, Taipei Medical University, Taipei 110, Taiwan; 103164@w.tmu.edu.tw; 5 Division of Cardiovascular Surgery, Department of Surgery, Wan Fang Hospital, Taipei Medical University, Taipei 116, Taiwan; 6 Graduate Institute of Biomedical Electronics and Bioinformatics, National Taiwan University, Taipei 10617, Taiwan; 7 Graduate Institute of Biomedical Informatics, College of Medical Science and Technology, Taipei Medical University, Taipei 110, Taiwan; 99356@w.tmu.edu.tw; 8 Artificial Intelligence Research and Development Center, Wan Fang Hospital, Taipei Medical University, Taipei 116, Taiwan; 9 Department of Radiation Oncology, Wan Fang Hospital, Taipei Medical University, Taipei 116, Taiwan; 10 Division of Nephrology, Department of Internal Medicine, Shuang Ho Hospital, Taipei Medical University, New Taipei City 235, Taiwan; 11 Department of Emergency and Critical Medicine, Wan Fang Hospital, Taipei Medical University, Taipei 116, Taiwan; 104228@w.tmu.edu.tw; 12 Department of Emergency Medicine, School of Medicine, College of Medicine, Taipei Medical University, Taipei 110, Taiwan; 13 Division of Cardiology and Cardiovascular Research Center, Department of Internal Medicine, Taipei Medical University Hospital, Taipei 110, Taiwan; 14 Division of Cardiology, Department of Medicine, Taipei Veterans General Hospital, Taipei 112, Taiwan; huangbs@vghtpe.gov.tw; 15 Second Degree Bachelor of Science in Nursing Collage of Medicine, National Taiwan University, Taipei 100, Taiwan; huilinghsieh@ntu.edu.tw; 16 Department of Nursing, National Taiwan University Hospital Yunlin Branch, Yunlin 640, Taiwan

**Keywords:** albuminuria, dulaglutide, glucagon like peptide 1 receptor agonist (GLP-1 RA), proteinuria, renal function, semaglutide

## Abstract

**Background:** GLP-1 receptor agonists (GLP-1 RAs) lower glucose and reduce cardiovascular events in type 2 diabetes, with noted renal benefits. Few studies directly compare GLP-1 RAs. This study aims to compare the effects of semaglutide and dulaglutide on renal function decline and proteinuria reduction in diabetic patients. **Methods:** The present study was conducted at Wanfang Hospital, Taipei Medical University. Diabetic patients using either semaglutide or dulaglutide for more than 1 year in the outpatient department from 1 January 2022 to 30 September 2024 were enrolled retrospectively. The outcome events in the present study included a decline in the estimated glomerular filtration rate (eGFR), an increase in the urine albumin–creatinine ratio (UACR), and patient death. **Results:** A total of 268 patients on dulaglutide and 747 on semaglutide were included. Baseline eGFR levels were similar in both groups. After 12 months, eGFR levels did not significantly decline in both groups. However, the dulaglutide group showed significantly higher UACR increases than the semaglutide group (*p* < 0.01). More death events also occurred in the dulaglutide group (*p* < 0.01). Multivariate logistic regression revealed a higher risk of UACR increase with dulaglutide (*p* < 0.01). Subgroup analysis found dulaglutide associated with higher UACR in patients younger than 60, males, those with hypertension, without heart failure, those using angiotensin receptor blockers, biguanides, and statins, and those not using sodium-glucose cotransporter-2 inhibitors. **Conclusions:** Dulaglutide and semaglutide had comparable effects on slowing eGFR decline. However, dulaglutide was less effective in reducing UACR, particularly in the subgroups mentioned above.

## 1. Introduction

Diabetes mellitus (DM) is one of the most significant chronic diseases, affecting approximately 451 million adults worldwide [1]. DM is associated with the accelerated progression of both macrovascular as well as microvascular complications [2]. Diabetic kidney disease (DKD) is a serious microvascular complication of diabetes, affecting approximately 40% of patients with DM [3]. Globally, the prevalence of DM in patients with end-stage kidney disease (ESKD) was 29.7% in 2015, and DM was responsible for 31.3% of incident ESKD cases in the same year [4]. In Taiwan, the prevalence of DKD among patients with type 2 DM was 17.92% in 2014, and it was responsible for approximately 45% ESKD cases [5]. On a global scale, DKD is the leading cause of ESKD, highlighting the critical importance of preventing and treating the renal complications of DM [6].

While glycemic and blood pressure control form the cornerstone of DKD treatment [7,8], several therapies have been specifically approved to slow renal function decline or reduce proteinuria in DM patients. From 1993 to early 2000, multiple clinical trials demonstrated that renin–angiotensin system blockade slows the renal decline in DKD by 5–7 mL/min/year, establishing its central role in DKD treatment [9,10,11]. Since then, progress in DKD treatment stagnated until 2014, when multiple trials reported the effect of sodium-glucose cotransporter 2 (SGLT2) inhibitors on DKD outcomes [12,13,14,15]. Around the same time, the nonsteroidal mineralocorticoid receptor antagonist finerenone was also shown to slow the progression of DKD [16]. We are now fortunate to be in a period of rapid advancements in DKD treatment and are about to witness the emergence of novel therapies for DKD.

Glucagon-like peptide 1 receptor agonists (GLP-1 RAs) are incretin-based antidiabetic agents that show a promising glucose-lowering effect in patients with type 2 DM [17,18,19]. In clinical trials, GLP-1 RAs have demonstrated a significant reduction in cardiovascular events associated with atherosclerosis. This evidence prompted a revision of treatment guidelines in 2020 [20,21,22]. The post hoc analysis of these trials indicated that semaglutide and liraglutide were linked to reductions in albuminuria and a decline in renal function [23]. A later published clinical trial, titled “Effect of Efpeglenatide on Cardiovascular Outcomes”, demonstrated that efpeglenatide significantly reduced the risk in composite kidney outcomes compared with a placebo [24]. Another GLP-1 RA, dulaglutide, was shown to marginally reduce the incidence of combined cardiovascular death and heart failure events in the REWIND trial [25]. The post hoc analysis indicated that dulaglutide may slow the progression of DKD, the decline in renal function, and the advancement to ESKD, suggesting its potential as an additional treatment option for DKD [26,27].

Semaglutide and dulaglutide are both GLP-1 RAs of human GLP-1 backbone that stimulate insulin secretion via the incretin effect, inhibit glucagon production to decrease the blood glucose level, delay gastric emptying to decrease calory intake, and prevent pancreatic β-cell apoptosis [28,29,30]. One difference between these two GLP-1 RAs may be that the clinical trials of semaglutide showed stronger effects of weight reduction and cardiovascular protection [31]. Recently, a meta-analysis of randomized controlled trials showed that GLP-1 RAs also significantly reduced adverse kidney events and renal failure, which make GLP-1 RAs an optimal treatment choice of DKD [32]. There is a scarcity of studies that perform direct head-to-head comparisons between GLP-1 RAs, which are less common in clinical trial settings. However, these comparisons can be crucial in formulating optimal strategies for the treatment of DKD. Therefore, this study aims to compare the renal effects of semaglutide and dulaglutide in a retrospective cohort of diabetic patients, focusing specifically on their impact on renal function decline and proteinuria reduction.

## 2. Methods

### 2.1. Study Design and Participants

The present study was conducted at Wanfang Hospital, Taipei Medical University. Diabetic patients using either semaglutide or dulaglutide for more than 1 year in the outpatient department from 1 January 2022 to 30 September 2024 were enrolled retrospectively. Medical records were reviewed to confirm eligibility for inclusion, demographic characteristics, comorbidities, laboratory data, and outcomes of interest. From the initiation of semaglutide or dulaglutide use, patients were followed for one year. Semaglutide was administered as Ozempic, manufactured by Novo Nordisk (Bagsværd, Gladsaxe Municipality, Denmark), at 0.25 mg to 1 mg per week subcutaneously. Dulaglutide was administered as Trulicity, manufactured by Lilly (Indianapolis, IN, USA), at 0.75 mg to 1.5 mg per week subcutaneously. The exclusion criteria were as follows: (1) the interchangeable use of semaglutide and dulaglutide, and (2) age under 20 years. The present study was approved by the Ethics Committee and Institutional Review Board of Taipei Medical University on 29 November 2024 (N202411053). The requirement for informed consent was waived. This study was conducted in accordance with the tenets of the 1975 Declaration of Helsinki, revised in 2000.

### 2.2. Definitions of Parameters and Outcomes

Demographic profile and comorbidities were defined at the initiation of semaglutide or dulaglutide use. The presence of hypertension was defined as the use of any antihypertensive medication at baseline. The presence of chronic kidney disease (CKD) was defined as an estimated glomerular filtration rate (eGFR) < 60 mL/min or a urine albumin–creatinine ratio (UACR) > 30 mcg/g for more than 3 months. The presence of DM was defined as a previous glycated hemoglobin (HbA1c) > 6.5% and the use of any antidiabetic medication. The attending cardiologist diagnosed heart failure (HF) based on either a reduced ejection fraction (EF) of less than 40% or a preserved EF that met the diagnostic criteria recommended by the Heart Failure Association of the European Society of Cardiology [33]. Laboratory data were collected at the baseline (the time of semaglutide or dulaglutide initiation) and the end (12 months after initiation). In the present study, eGFR was calculated using the CKD-EPI equation, revised in 2021 [34]. The medications of interest that were co-administered included sodium-glucose transport protein 2 (SGLT2) inhibitors, biguanides, furosemide, angiotensin-converting enzyme inhibitors (ACEIs)/angiotensin receptor blockers (ARBs), spironolactone, thiazide, and statin. The use of a specific medication was defined as its prescription for a duration exceeding three months within the study period.

The difference between the end eGFR and the baseline eGFR was termed eGFR change. The difference between the end UACR and baseline UACR was termed UACR change. The outcome events in the present study included eGFR decline defined as the decrease in eGFR for more than 10% from its baseline, UACR increase defined as the increase in UACR for more than 10% from its baseline, and patient death by the 1-year study period.

### 2.3. Statistical Analyses

Continuous variables with a normal distribution were reported as mean ± standard deviation, while continuous variables that deviated from a normal distribution were expressed as medians (25th and 75th percentiles). Categorical variables were reported as frequency and percentage. Statistical analyses of continuous variables with a normal distribution were conducted using Student’s *t*-test for independent samples, analyses of continuous variables that deviated from a normal distribution were conducted using the Wilcoxon signed-rank test, and analyses of categorical variables were conducted using the chi square test. The changes in values between the baseline and the end of the study were analyzed using a dependent *t*-test for paired samples. The association between parameters and the outcomes was evaluated using logistic regression methods. Each parameter was pre-evaluated for its association with the outcomes using univariate logistic regression. Parameters significantly associated with the outcomes (*p* < 0.05) were included in the multivariate logistic regression model. Missing values were imputed using the last observation carried forward method. Statistical analysis was performed using SAS 9.4 (SAS Institute Inc., Cary, NC, USA).

## 3. Results

### 3.1. Demographic and Clinical Baseline Characteristics of Patients

During the data collection period, 268 patients using dulaglutide and 747 patients using semaglutide were eligible for inclusion in the study. Among all of the study patients, the mean age was 59.5 ± 14.2 years and 55.5% were male. Additionally, 71.2% had hypertension, 62.6% had chronic kidney disease (CKD), 12.9% had heart failure (HF), and 72.5% had dyslipidemia. Compared to the patients on semaglutide, the patients using dulaglutide were significantly older, included more males, and had a higher prevalence of hypertension. At baseline, the mean SCr was 0.9 ± 0.5 mg/dL and the mean eGFR was 90.0 ± 31.7 mL/min/1.73 m^2^. There were no significant differences in the baseline SCr and eGFR between the patients on dulaglutide and those on semaglutide. Patients on dulaglutide exhibited significantly higher HbA1c levels, lower serum albumin, and higher serum triglycerides. At baseline, the median UACR was 50.0 mcg/g in patients using dulaglutide and 65.0 mcg/g in patients on semaglutide, with no significant difference between the two groups (Table 1).

Out of the total 1015 patients included in the study, 21.9% were co-administered with SGLT2 inhibitors. Patients on dulaglutide had a significantly higher rate of co-administration with SGLT2 inhibitors compared to patients on semaglutide (34.3% versus 17.5%, *p* < 0.01). Regarding medications blocking the renin–angiotensin–aldosterone axis, 56.0% and 3.8% of the total included patients were using ARB and spironolactone, respectively, with no significant difference between the two groups. Additionally, patients on dulaglutide had significantly higher rates of co-administration of biguanides and statins, while the use of loop diuretics and thiazides was similar between the two groups (Table 2).

### 3.2. Comparative Analysis of Renal Outcomes in Patients Treated with Dulaglutide Versus Semaglutide

**Figure 1:**
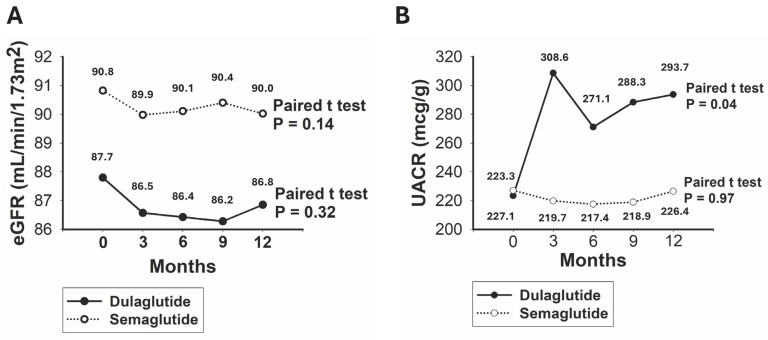
Evolving outcome values throughout the study duration. (**A**) Visualization of eGFR trends; (**B**) visualization of UACR trends. eGFR, estimated glomerular filtration rate; UACR, urine albumin–creatinine ratio. The changes in values between the baseline and the end of the study were analyzed using a dependent *t*-test for paired samples.

The baseline eGFR was 87.7 mL/min/1.72 m^2^ for the dulaglutide group and 90.8 mL/min/1.72 m^2^ for the semaglutide group, with no significant difference between the two groups (*p* = 0.20). For patients on dulaglutide, the mean eGFR was 86.8 mL/min/1.72 m^2^ after 12 months. The mean eGFR at baseline and after 12 months did not differ significantly (*p* = 0.32). For patients on semaglutide, the mean eGFR was 90.0 mL/min/1.72 m^2^ after 12 months, with no significant difference between the baseline and 12-month values (*p* = 0.13) (Figure 1A). The baseline UACR was 223.3 mcg/g for the dulaglutide group and 227.1 mcg/g for the semaglutide group, showing no significant difference between the two groups (*p* = 0.94). After 12 months, the mean UACR for the dulaglutide group increased to 293.7 mcg/g, which was significantly higher than the baseline value (*p* = 0.04). For the semaglutide group, the mean UACR was 226.4 mcg/g after 12 months, with no significant difference from the baseline value (*p* = 0.97) (Figure 1B).

In total, the mean eGFR change was −0.8 mL/min, and 243 (23.9%) of the 1015 included patients experienced an eGFR decline after 12 months of using GLP-1 RA. In the dulaglutide group, 25.7% of patients had an eGFR decline, while, in the semaglutide group, 23.2% of patients experienced an eGFR decline. The mean value of the eGFR change and the frequencies of the events of eGFR decline were not significantly different between the two groups. On the other hand, 220 (21.6%) of all included patients experienced a UACR increase, and the mean UACR change was 18.1 mcg/g after 12 months. In the dulaglutide group, the mean UACR change was 70.3 mcg/g and 36.5% of patients had a UACR increase, whereas in the semaglutide group, the mean UACR change was −0.6 mcg/g and 19.4% of patients had an increase. The frequency of a UACR increase event and the mean UACR change were significantly higher in the dulaglutide group (Table 3).

The above finding suggests that the dulaglutide group may have a higher chance of experiencing a UACR increase compared to the semaglutide group. To confirm this, a multivariate logistic regression model, adjusted for factors associated with an increased UACR, was constructed. Candidate risk factors significantly associated with the UACR increase in univariate logistic regression (*p* < 0.05) were included in the multivariate logistic regression model. These factors include the use of dulaglutide or semaglutide, age, gender, hypertension, CKD, dyslipidemia, baseline HbA1c, and serum low-density lipoprotein cholesterol (LDL), and the use of SGLT2 inhibitors, angiotensin II receptor blockers (ARBs), biguanides, and statins. In the multivariate logistic regression model, the dulaglutide group exhibited a significantly higher risk for a UACR increase compared to the semaglutide group, with an odds ratio (OR) of 1.6 and a 95% confidence interval (CI) of 1.2–2.3 (*p* < 0.01). Other significant risk factors for a UACR increase included male gender, hypertension, higher HbA1c, higher serum albumin, and the use of SGLT2 inhibitors (Table 4).

### 3.3. UACR Subgroup Outcomes in Patients Using Dulaglutide Versus Semaglutide

**Figure 2:**
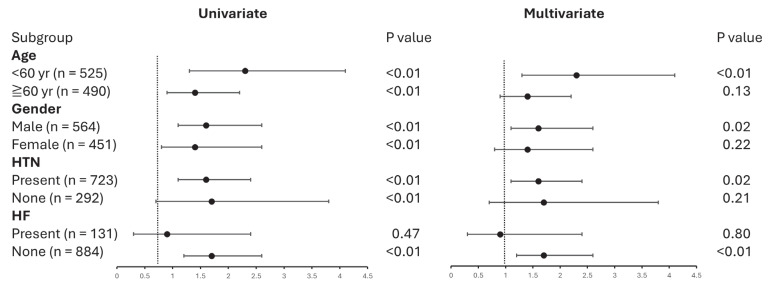
Subgroup analysis on the risk of UACR in patients treated with dulaglutide versus semaglutide. HTN, hypertension; HF, heart failure. Statistical analyses were performed by using logistic regression. The establishment of the multivariate logistic regression models are referred to in Supplementary Tables S1–S8.

To elucidate the impacts of the identified risk factors on the association between dulaglutide and semaglutide use on UACR, subgroup analyses were conducted based on age, gender, the presence of hypertension, and HF. Among patients under 60 years of age, the dulaglutide group had a significantly higher risk of a UACR increase compared to the semaglutide group. In contrast, for patients aged 60 years and older, the dulaglutide group did not exhibit a significant risk of UACR increase compared to the semaglutide group. In male patients, the dulaglutide group exhibited a significantly higher risk of UACR increase compared to the semaglutide group. Conversely, in female patients, the dulaglutide group did not show a significantly higher risk of UACR increase compared to the semaglutide group. In patients with hypertension, the dulaglutide group showed a significantly higher risk for UACR increase compared to the semaglutide group. Conversely, in patients without hypertension, the dulaglutide group did not have a significantly higher risk for UACR increase compared to the semaglutide group. In patients with HF, there was no significant difference in the risk of UACR increase between the dulaglutide and semaglutide groups. Notably, in patients without HF, the dulaglutide group showed a significantly higher risk for UACR increase compared to the semaglutide group. These findings suggest that age, gender, and the presence of hypertension and HF may interact with GLP-1 RAs in their effect on UACR increase (Figure 2). The construction of the multivariate logistic regression models are referred to in Supplementary Tables S1–S8.

**Figure 3:**
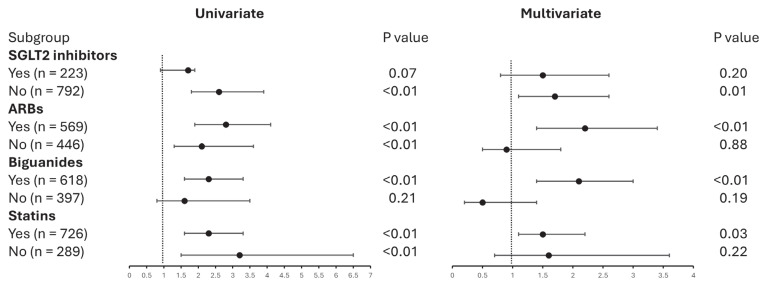
Impact of medication combinations on UACR risk in patients treated with dulaglutide versus semaglutide. SGLT2, sodium-glucose cotransporter-2; ARB, angiotensin receptor blockers. Statistical analyses were performed by using logistic regression. The establishment of the multivariate logistic regression models are referred to Supplementary Tables S9–S16.

To elucidate the interactions between dulaglutide/semaglutide use and other medications in the multivariate logistic regression model, subgroup analyses were conducted based on the use of SGLT2 inhibitors, ARBs, biguanides, and statins. For the users of SGLT2 inhibitors, the dulaglutide group did not show a significant risk for UACR increase compared to the semaglutide group. In contrast, for nonusers of SGLT2 inhibitors, the dulaglutide group exhibited a significantly higher risk for UACR increase compared to the semaglutide group. Among ARB users, the dulaglutide group showed a significantly higher risk for UACR increase compared to the semaglutide group. However, in nonusers of ARB, the dulaglutide group did not show a significantly higher risk for UACR increase compared to the semaglutide group. For biguanide users, the dulaglutide group exhibited a significantly higher risk for UACR increase compared to the semaglutide group. Conversely, among nonusers of biguanides, the dulaglutide group did not demonstrate a significantly higher risk for UACR increase compared to the semaglutide group. Among statin users, the dulaglutide group exhibited a significantly higher risk for UACR increase compared to the semaglutide group. However, for nonusers of statins, the dulaglutide group did not show a significantly higher risk for UACR increase compared to the semaglutide group (Figure 3). The construction of multivariate logistic regression models are referred to in Supplementary Tables S9–S16.

## 4. Discussion

In summary, the main findings of the present study indicate that patients using dulaglutide experienced a significant increase in UACR after 12 months of treatment, whereas patients using semaglutide maintained similar UACR levels after the same period. Additionally, patients using dulaglutide exhibited a significantly higher UACR change, and more events of UACR increase and death, than the patients using semaglutide. The multivariate logistic regression model showed that the dulaglutide group had a significantly higher risk for UACR increase compared to the semaglutide group. Subgroup analysis revealed that the relationship between dulaglutide/semaglutide use and the higher risk of UACR increase remained consistent in patients aged younger than 60 years, male patients, patients with hypertension, patients without heart failure, patients using ARBs, biguanides, and statins, and patients not using SGLT2 inhibitors.

Previous studies have demonstrated the renal protective effects of GLP-1 RAs. A pooled analysis of clinical trials involving semaglutide revealed that semaglutide significantly slowed the decline in eGFR and the progression of UACR compared to placebo, especially in patients with pre-existing CKD [23,35]. Regarding dulaglutide, a post hoc analysis indicated that dulaglutide treatment was associated with fewer events of eGFR decline. However, it did not show a significant difference in the UACR compared to placebo [27]. In a retrospective study with a small sample size, dulaglutide was associated with a reduced decline in eGFR in patients with CKD [36]. The findings from these previous studies indicate that both dulaglutide and semaglutide may offer protective benefits against the decline in eGFR. However, only semaglutide appears to have protective effects against increases in the UACR, though further evidence is needed to confirm this. In response to these findings, the present study conducted a head-to-head comparison and demonstrated that dulaglutide and semaglutide exhibit similar protective effects in eGFR decline. However, semaglutide showed a significantly greater effect in reducing increases in UACR than did dulaglutide.

A recent meta-analysis comparing the efficacy of dulaglutide and semaglutide showed that both medications have similar efficacy in glycemic control. However, semaglutide demonstrated higher efficacy in inducing weight loss, and is therefore more suitable for patients requiring both weight reduction and glycemic control [37]. Additionally, an indirect treatment comparison demonstrated significantly greater reductions from baseline in HbA1c and body weight with semaglutide compared to dulaglutide [38]. Furthermore, a post hoc analysis suggested that semaglutide demonstrated superior efficacy compared to dulaglutide in terms of glycemic control and body weight reduction, irrespective of gender, diabetes duration, and baseline body mass index (BMI) [39]. Despite these findings, there are limited studies comparing which patient subgroups are most suitable for dulaglutide versus semaglutide based on age, gender, or comorbidities. The present study found that dulaglutide was associated with a higher UACR compared to semaglutide in patients who were younger, male, hypertensive, and without HF. This suggests that dulaglutide may be more favorable for patients who are older, female, non-hypertensive, and those with HF. These novel findings may help clinicians make individualized selections of GLP-1 RAs for patients.

Several studies have shown that the combined use of SGLT2 inhibitors and GLP-1 receptor agonists is associated with a reduction in major cardiovascular events and serious renal events in patients with type 2 DM [40,41,42]. However, studies investigating the comparative effects of dulaglutide versus semaglutide in combination with other medications remain absent to date. The present study showed that dulaglutide was associated with a higher risk of UACR increase versus semaglutide in the case of the combination with ARBs, biguanides, and statins, and without the combination of SGLT2 inhibitors. These findings suggest that dulaglutide may be a preferable option for patients not using ARBs, biguanides, and statins, and those who are using SGLT2 inhibitors. In contrast, semaglutide may be preferable in patients using ARBs, biguanides, and statins, and patients who are not using SGLT2 inhibitors.

The limitations of the present study include its single-center base, retrospective design, relatively small sample size, possible selection bias, lack of randomization, absent data on protein intake, and blood pressure monitoring during the follow-up period. To compensate for these limitations, the study ensured similar baseline eGFR and UACR levels between the dulaglutide and semaglutide groups, allowing for a comparative analysis of these outcomes. Additionally, a multivariate logistic method was employed to adjust for uncontrolled variables. While the mechanism underlying the renal effects of GLP-1 receptor agonists remains unclear, further investigation is needed to determine the differences in their impact on UACR reduction between semaglutide and dulaglutide.

In conclusion, the present study showed that dulaglutide was associated with a significantly higher UACR compared to semaglutide, especially in patients of younger age, male gender, with hypertension, without HF, those using ARBs, biguanides, and statins, and those who are not using SGLT2 inhibitors. These findings may help clinicians make individualized selections of GLP-1 RAs for diabetic patients.

## Figures and Tables

**Table 1:** Baseline characteristics of the study patients.

Character	Total	Dulaglutide	Semaglutide	*p* Value
Number, *n*	1015	268	747	n/a
Age, yr	59.5 ± 14.2	63.6 ± 15.1	58.0 ± 13.6	<0.01
Male, *n* (%)	564 (55.5)	163 (60.8)	401 (53.6)	0.04
HTN, *n* (%)	723 (71.2)	208 (77.6)	515 (68.9)	<0.01
CKD, *n* (%)	636 (62.6)	169 (63.0)	467 (62.5)	0.87
HF, *n* (%)	131 (12.9)	43 (16.0)	88 (11.7)	0.07
Dyslipidemia, *n* (%)	736 (72.5)	195 (72.7)	541 (72.4)	0.91
Creatinine, mg/dL	0.9 ± 0.5	1.0 ± 0.7	0.9 ± 0.5	0.08
eGFR, mL/min/1.73 m^2^	90.0 ± 31.7	87.7 ± 34.2	90.8 ± 30.8	0.20
HbA1c, %	7.7 ± 1.8	8.7 ± 1.7	7.3 ± 1.7	<0.01
Albumin, g/dL	3.8 ± 0.4	3.6 ± 0.5	3.8 ± 0.3	<0.01
ALT, U/L	20.0 (17.0)	21.0 (20.0)	20.0 (17.0)	0.09
LDL, mg/dL	90.4 ± 31.3	91.6 ± 31.8	90.0 ± 31.1	0.47
TRIG, mg/dL	136.0 (93.0)	150.5 (114.5)	132.0 (90.0)	<0.01
UACR, mg/g	50.0 (88.0)	50.0 (133.0)	65.0 (88.0)	0.98

Yr, year; HTN, hypertension; CKD, chronic kidney disease; eGFR, estimated glomerular filtration rate; HbA1c, glycated hemoglobin; ALT, alanine aminotransferase; LDL, low-density lipoprotein; TRIG, triglyceride; UACR, urine albumin-to-creatinine ratio; n/a, not applicable. Category variables were analyzed using chi square test. Continuous variables with normal distribution were expressed as mean ± standard deviation and analyzed using Student’s *t*-test for independent samples. Continuous variables deviated from normal distribution were expressed as median (interquartile range) and analyzed using Wilcoxon rank sum test.

**Table 2:** Medication profiles of the study patients.

Character	Total	Dulaglutide	Semaglutide	*p* Value
Number, *n*	1015	268	747	n/a
SGLT2 inhibitor, *n* (%)	223 (21.9)	92 (34.3)	131 (17.5)	<0.01
ARB, *n* (%)	569 (56.0)	156 (58.2)	413 (55.2)	0.40
Spironolactone, *n* (%)	39 (3.8)	14 (5.2)	25 (3.3)	0.17
Biguanides, *n* (%)	618 (60.8)	205 (76.4)	413 (55.2)	<0.01
Loop diuretics, *n* (%)	43 (4.2)	15 (5.6)	28 (3.7)	0.19
Thiazide, *n* (%)	94 (9.2)	24 (8.9)	70 (9.3)	0.84
Statins, *n* (%)	726 (71.5)	205 (76.4)	521 (69.7)	0.03

SGLT2, sodium-glucose cotransporter-2; ARB, angiotensin receptor blockers; n/a, not applicable. Category variables were analyzed using chi square test.

**Table 3:** One-year renal outcomes of dulaglutide versus semaglutide treatment.

Outcomes	Total	Dulaglutide	Semaglutide	*p* Value
eGFR change, mL/min	−0.8 ± 0.4	−0.9 ± 0.9	−0.8 ± 0.5	0.69
eGFR decline, *n* (%)	243 (23.9)	69 (25.7)	174 (23.2)	0.41
UACR change, mcg/g	18.1 ± 15.9	70.3 ± 34.7	−0.6 ± 17.7	<0.01
UACR increase, *n* (%)	220 (21.6)	92 (34.3)	128 (17.1)	<0.01
Death, *n* (%)	8 (0.8)	6 (2.2)	2 (0.2)	<0.01

eGFR, estimated glomerular filtration rate; UACR, urine albumin-to-creatinine ratio. Event of eGFR decline was defined as eGFR decrease of more than 10% from baseline eGFR; event of UACR increase was defined as UACR increase of more than 10% from baseline UACR. Continuous variables were analyzed using Student’s *t*-test or Wilcoxon rank sum test, as appropriate. Categorical variables were analyzed using chi square method.

**Table 4:** Comparative analysis of the risk of UACR increase in patients treated with dulaglutide versus semaglutide.

Character	Univariate			Multivariate		
	**OR**	**95% CI**	***p* Value**	**OR**	**95% CI**	***p* Value**
Dulaglutide use	2.4	1.8–3.3	<0.01	1.6	1.1–2.3	<0.01
Age, per 10 yr increment	1.3	1.2–1.4	<0.01	1.2	1.1–1.4	<0.01
Male	1.6	1.2–2.2	<0.01	1.2	0.9–1.7	0.22
HTN	2.0	1.4–2.9	<0.01	1.4	0.9–2.1	0.11
CKD	1.5	1.1–2.2	0.02	0.8	0.5–1.1	0.10
HF	0.6	0.4–0.9	0.04	0.6	0.3–0.9	0.03
Dyslipidemia	1.5	1.1–2.1	0.02	1.0	0.7–1.5	0.84
HbA1c, per 1% increment	1.3	1.2–1.4	<0.01	1.2	1.1–1.3	<0.01
Albumin, per 1 g/dL increment	1.1	0.8–1.5	0.58			
ALT, per 30 U/L increment	0.8	0.7–1.1	0.20			
LDL, per 100 mg/dL increment	0.5	0.3–0.8	<0.01	0.6	0.3–1.0	0.07
TRIG, per 100 mg/dL increment	1.0	0.9–1.0	0.26			
SGLT2 inhibitors use	2.1	1.5–2.9	<0.01	1.7	1.2–2.4	<0.01
ARB use	1.8	1.3–2.4	<0.01	1.1	0.7–1.6	0.56
Spironolactone use	1.1	0.5–2.2	0.79			
Biguanides use	3.4	2.4–4.9	<0.01	1.5	0.9–2.3	0.08
Loop diuretics use	1.2	0.6–2.4	0.53			
Thiazide use	1.2	0.7–1.9	0.37			
Statin use	2.3	1.6–3.3	<0.01	1.3	0.8–2.0	0.22

OR, odds ratio; CI, confidence interval; Yr, year; HTN, hypertension; CKD, chronic kidney disease; HF, heart failure; HbA1c, glycated hemoglobin; ALT, alanine aminotransferase; LDL, low-density lipoprotein; TRIG, triglyceride; SGLT2, sodium-glucose cotransporter-2; ARB, angiotensin receptor blockers; UACR, urine albumin-to-creatinine ratio. Risk analysis performed by using logistic regression model. Variables with *p* values of <0.05 in univariate logistic regression models were included into the multivariate logistic regression model.

## Data Availability

The data that support the findings of this study are available from the corresponding author, Chung-Te Liu, upon reasonable request.

## Supplementary Materials

The following supporting information can be downloaded at: https://www.mdpi.com/article/10.3390/jcm14124287/s1, Table S1: Subgroup Analysis of the Risk of UACR Increase in Patients Under 60 Years of Age; Table S2: Subgroup Analysis of the Risk of UACR Increase in Patients Above 60 Years of Age; Table S3: Subgroup Analysis of the Risk of UACR Increase in Male Patients; Table S4: Subgroup Analysis of the Risk of UACR Increase in Female Patients; Table S5: Subgroup Analysis of the Risk of UACR Increase in Patients with Hypertension; Table S6: Subgroup Analysis of the Risk of UACR Increase in Patients without Hypertension; Table S7: Subgroup Analysis of the Risk of UACR Increase in Patients with HF; Table S8: Subgroup Analysis of the Risk of UACR Increase in Patients without HF; Table S9: Subgroup Analysis of the Risk of UACR Increase in Patients Using SGLT2 inhibitors; Table S10: Subgroup Analysis of the Risk of UACR Increase in Patients Not Using SGLT2 inhibitors; Table S11: Subgroup Analysis of the Risk of UACR Increase in Patients Using ARBs; Table S12: Subgroup Analysis of the Risk of UACR Increase in Patients Not Using ARBs; Table S13: Subgroup Analysis of the Risk of UACR Increase in Patients Using Biguanides; Table S14: Subgroup Analysis of the Risk of UACR Increase in Patients Not Using Biguanides; Table S15: Subgroup Analysis of the Risk of UACR Increase in Patients Using Statins; Table S16: Subgroup Analysis of the Risk of UACR Increase in Patients Not Using Statins.
