# Supplementary material for: Comparative Effects of Dulaglutide and Semaglutide on Renal Function Decline and Proteinuria Reduction in Diabetic Patients: A Retrospective Cohort Study
Source: J Clin Med. 2025 Jun 16;14(12):4287. doi: 10.3390/jcm14124287 (PMC12193715; doi:10.3390/jcm14124287)
Supplement: Supplementary file 1 [file jcm-14-04287-s001.zip › jcm-3625387-supplementary.pdf]

Supplementary Table S1. Subgroup Analysis of the Risk of UACR Increase in Patients Under 60 Years of Age.

Character	Univariate			Multivariate		
	OR	95% CI	P value	OR	95% CI	P value
Dulaglutide use	3.2	1.9–5.5	<.01	2.3	1.3–4.1	<.01
Age, per 10 yr increment	1.3	1.1–1.8	0.04	1.3	0.9–1.8	0.14
Male	1.8	1.1–3.0	0.02	1.4	0.8–2.4	0.29
HTN	1.3	0.7–2.2	0.27			
CKD	0.4	0.3–0.7	<.01	1.4	0.7–2.4	0.40
HF	0.9	0.4–2.6	0.96			
Dyslipidemia	1.3	0.8–2.3	0.30			
HbA1c, per 1% increment	24.0	7.1–81.0	<.01	1.2	1.1–1.4	0.03
Albumin, per 1 g/dL increment	0.9	0.5–1.8	0.91			
ALT, per 30 U/L increment	0.8	0.7–1.2	0.42			
LDL, per 100 mg/dL increment	0.4	0.2–0.9	0.04	0.6	0.2–1.5	0.26
TRIG, per 100 mg/dL increment	1.0	0.9–1.1	0.34			
SGLT2 inhibitors use	3.2	1.8–5.4	<.01	2.1	1.1–3.7	0.01
ARB use	2.0	1.2–3.3	<.01	1.2	0.7–2.2	0.45
Spironolactone use	0.5	0.1–4.1	0.53			
Biguanides use	5.6	2.9–10.7	<.01	2.4	1.1–5.3	0.02
Loop diuretics use	2.7	0.7–11.0	0.16			
Thiazide use	1.4	0.6–3.1	0.41			
Statin use	2.3	1.3–4.2	<.01	0.9	0.4–1.8	0.75

OR, odds ratio; CI, confidence interval; Yr, year; HTN, hypertension; CKD, chronic kidney disease; HF, heart failure; HbA1c, glycated hemoglobin; ALT, alanine aminotransferase; LDL, low density lipoprotein; TRIG, triglyceride; SGLT2, sodium-glucose cotransporter-2; ARB, angiotensin receptor blockers; UACR, urine albumin-to-creatinine ratio.

Risk analysis performed by using logistic regression model. Variables with P values of < 0.05 in univariate logistic regression models were included into the multivariate logistic regression model.

Supplementary Table S2. Subgroup Analysis of the Risk of UACR Increase in Patients Above 60 Years of Age.

Character	Univariate			Multivariate		
	OR	95% CI	P value	OR	95% CI	P value
Dulaglutide use	1.9	1.3–2.9	<.01	1.4	0.9–2.2	0.13
Age, per 10 yr increment	1.3	0.9–1.6	0.06			
Male	1.3	0.9–1.9	0.13			
HTN	2.1	1.2–3.7	<.01	0.6	1.3–3.9	<.01
CKD	0.9	0.6–1.4	0.84			
HF	0.5	0.3–0.9	0.03	0.6	0.3–1.0	0.05
Dyslipidemia	1.4	0.8–2.2	0.20			
HbA1c, per 1% increment	1.3	1.1–1.4	<.01	1.2	1.1–1.4	<.01
Albumin, per 1 g/dL increment	0.9	0.6–1.4	0.79			
ALT, per 30 U/L increment	0.8	0.6–1.2	0.28			
LDL, per 100 mg/dL increment	0.7	0.4–1.5	0.44			
TRIG, per 100 mg/dL increment	1.1	0.9–1.3	0.13			
SGLT2 inhibitors use	1.8	1.2–2.9	<.01	1.5	0.9–2.5	0.05
ARB use	1.5	0.9–2.2	0.07			
Spironolactone use	1.3	0.6–2.9	0.53			
Biguanides use	1.9	1.3–3.1	<.01	1.3	0.8–2.1	0.34
Loop diuretics use	1.3	0.6–2.8	0.47			
Thiazide use	1.1	0.6–2.1	0.71			
Statin use	1.9	1.2–3.2	0.01	1.6	0.9–2.8	0.08

OR, odds ratio; CI, confidence interval; Yr, year; HTN, hypertension; CKD, chronic kidney disease; HF, heart failure; HbA1c, glycated hemoglobin; ALT, alanine aminotransferase; LDL, low density lipoprotein; TRIG, triglyceride; SGLT2, sodium-glucose cotransporter-2; ARB, angiotensin receptor blockers; UACR, urine albumin-to-creatinine ratio.

Risk analysis performed by using logistic regression model. Variables with P values of < 0.05 in univariate logistic regression models were included into the multivariate logistic regression model.

Supplementary Table S3. Subgroup Analysis of the Risk of UACR Increase in Male Patients.

Character	Univariate			Multivariate		
	OR	95% CI	P value	OR	95% CI	P value
Dulaglutide use	2.7	1.8–4.0	<.01	1.6	1.1–2.6	0.02
Age, per 10 yr increment	1.3	1.2–1.6	<.01	1.3	1.1–1.5	<.01
HTN	1.4	0.9–2.3	0.13			
CKD	0.9	0.7–1.5	0.91			
HF	0.9	0.5–1.6	0.71			
Dyslipidemia	1.4	0.9–2.3	0.15			
HbA1c, per 1% increment	1.3	1.2–1.5	<.01	1.2	1.1–1.4	<.01
Albumin, per 1 g/dL increment	0.9	0.6–1.5	0.90			
ALT, per 30 U/L increment	0.7	0.5–1.0	0.05			
LDL, per 100 mg/dL increment	0.5	0.3–1.0	0.05			
TRIG, per 100 mg/dL increment	1.1	0.9–1.2	0.28			
SGLT2 inhibitors use	2.5	1.6–3.7	<.01	2.0	1.3–3.0	<.01
ARB use	1.2	0.8–1.8	0.40			
Spironolactone use	1.0	0.4–2.5	0.92			
Biguanides use	2.7	1.7–4.3	<.01	1.6	0.9–2.9	0.09
Loop diuretics use	3.0	1.3–7.0	0.01	2.8	1.1–7.4	0.03
Thiazide use	1.3	0.7–2.4	0.43			
Statin use	2.3	1.4–4.0	<.01	1.8	0.9–3.2	0.05

OR, odds ratio; CI, confidence interval; Yr, year; HTN, hypertension; CKD, chronic kidney disease; HF, heart failure; HbA1c, glycated hemoglobin; ALT, alanine aminotransferase; LDL, low density lipoprotein; TRIG, triglyceride; SGLT2, sodium-glucose cotransporter-2; ARB, angiotensin receptor blockers; UACR, urine albumin-to-creatinine ratio.

Risk analysis performed by using logistic regression model. Variables with P values of < 0.05 in univariate logistic regression models were included into the multivariate logistic regression model.

Supplementary Table S4. Subgroup Analysis of the Risk of UACR Increase in Female Patients.

Character	Univariate			Multivariate		
	OR	95% CI	P value	OR	95% CI	P value
Dulaglutide use	2.2	1.3–3.7	<.01	1.4	0.8–2.6	0.22
Age, per 10 yr increment	1.3	1.1–1.6	<.01	1.2	0.9–1.5	0.05
HTN	2.7	1.5–5.0	<.01	1.5	0.9–3.1	0.26
CKD	0.5	0.3–0.8	<.01	0.5	0.3–0.8	<.01
HF	0.5	0.2–1.3	0.15			
Dyslipidemia	1.4	0.8–2.5	0.18			
HbA1c, per 1% increment	1.3	1.1–1.5	<.01	1.2	0.9–1.3	0.06
Albumin, per 1 g/dL increment	0.9	0.5–1.5	0.61			
ALT, per 30 U/L increment	0.8	0.5–1.2	0.26			
LDL, per 100 mg/dL increment	0.5	0.2–1.1	0.09			
TRIG, per 100 mg/dL increment	1.0	0.9–1.1	0.49			
SGLT2 inhibitors use	2.0	1.1–3.6	0.01	1.3	0.7–2.5	0.37
ARB use	3.1	1.8–5.2	<.01	1.9	1.0–3.2	0.04
Spironolactone use	1.7	0.4–6.5	0.44			
Biguanides use	3.4	1.9–5.9	<.01	1,7	0.8–3.3	0.14
Loop diuretics use	0.7	0.2–2.7	0.68			
Thiazide use	1.2	0.5–2.7	0.66			
Statin use	1.9	1.1–3.3	0.01	0.9	0.5–1.7	0.73

OR, odds ratio; CI, confidence interval; Yr, year; HTN, hypertension; CKD, chronic kidney disease; HF, heart failure; HbA1c, glycated hemoglobin; ALT, alanine aminotransferase; LDL, low density lipoprotein; TRIG, triglyceride; SGLT2, sodium-glucose cotransporter-2; ARB, angiotensin receptor blockers; UACR, urine albumin-to-creatinine ratio.

Risk analysis performed by using logistic regression model. Variables with P values of < 0.05 in univariate logistic regression models were included into the multivariate logistic regression model.

Supplementary Table S5. Subgroup Analysis of the Risk of UACR Increase in Patients with Hypertension.

Character	Univariate			Multivariate		
	OR	95% CI	P value	OR	95% CI	P value
Dulaglutide use	2.4	1.7–3.4	<.01	1.6	1.1–2.4	0.02
Age, per 10 yr increment	1.4	1.2–1.6	<.01	1.3	1.1–1.5	<.01
Male	1.2	0.8–1.7	0.30			
CKD	0.8	0.6–1.2	0.33			
HF	0.7	0.4–1.1	0.11			
Dyslipidemia	1.2	0.8–1.8	0.41			
HbA1c, per 1% increment	1.3	1.2–1.4	<.01	1.2	1.1–1.3	<.01
Albumin, per 1 g/dL increment	0.9	0.6–1.3	0.47			
ALT, per 30 U/L increment	0.7	0.5–0.9	0.01	0.8	0.6–1.0	0.07
LDL, per 100 mg/dL increment	0.6	0.4–1.0	0.10			
TRIG, per 100 mg/dL increment	1.0	0.9–1.1	0.45			
SGLT2 inhibitors use	2.0	1.4–2.9	<.01	1.6	1.1–2.4	0.02
ARB use	1.2	0.8–1.7	0.34			
Spironolactone use	1.0	0.4–2.3	0.94			
Biguanides use	2.2	1.5–3.3	<.01	1.4	0.9–2.4	0.11
Loop diuretics use	1.8	0.9–3.5	0.09			
Thiazide use	1.0	0.6–1.7	0.85			
Statin use	1.9	1.2–2.9	<.01	1.5	0.9–2.4	0.12

OR, odds ratio; CI, confidence interval; Yr, year; CKD, chronic kidney disease; HF, heart failure; HbA1c, glycated hemoglobin; ALT, alanine aminotransferase; LDL, low density lipoprotein; TRIG, triglyceride; SGLT2, sodium-glucose cotransporter-2; ARB, angiotensin receptor blockers; UACR, urine albumin-to-creatinine ratio.

Risk analysis performed by using logistic regression model. Variables with P values of < 0.05 in univariate logistic regression models were included into the multivariate logistic regression model.

Supplementary Table S6. Subgroup Analysis of the Risk of UACR Increase in Patients without Hypertension.

Character	Univariate			Multivariate		
	OR	95% CI	P value	OR	95% CI	P value
Dulaglutide use	2.8	1.4–5.6	<.01	1.7	0.7–3.8	0.21
Age, per 10 yr increment	1.1	0.8–1.3	0.65			
Male	2.3	1.2–4.6	0.01	1.2	0.6–2.7	0.60
CKD	0.4	0.2–0.7	<.01	0.6	0.3–1.3	0.17
HF	1.0	0.3–3.1	0.98			
Dyslipidemia	2.5	1.1–5.7	0.02	1.5	0.6–4.0	0.38
HbA1c, per 1% increment	1.3	1.2–1.6	<.01	1.2	0.9–1.5	0.22
Albumin, per 1 g/dL increment	1.3	0.6–2.8	0.47			
ALT, per 30 U/L increment	1.0	0.7–1.5	0.76			
LDL, per 100 mg/dL increment	0.3	0.1–0.9	0.04	0.8	0.5–1.3	0.33
TRIG, per 100 mg/dL increment	1.0	0.9–1.2	0.60			
SGLT2 inhibitors use	3.9	1.9–28.0	<.01	2.0	1.1–5.1	0.09
ARB use	3.3	1.7–6.6	<.01	2.4	1.1–5.1	0.02
Spironolactone use	2.4	0.4–12.7	0.30			
Biguanides use	13.6	4.1–45.3	<.01	5.4	1.4–20.7	0.01
Loop diuretics use	1.8	0.9–3.5	0.98			
Thiazide use	2.4	0.4–12.7	0.30			
Statin use	2.6	1.2–5.7	0.01	0.7	0.7–2.1	0.60

OR, odds ratio; CI, confidence interval; Yr, year; CKD, chronic kidney disease; HF, heart failure; HbA1c, glycated hemoglobin; ALT, alanine aminotransferase; LDL, low density lipoprotein; TRIG, triglyceride; SGLT2, sodium-glucose cotransporter-2; ARB, angiotensin receptor blockers; UACR, urine albumin-to-creatinine ratio.

Risk analysis performed by using logistic regression model. Variables with P values of < 0.05 in univariate logistic regression models were included into the multivariate logistic regression model.

Supplementary Table S7. Subgroup Analysis of the Risk of UACR Increase in Patients with HF.

Character	Univariate			Multivariate		
	OR	95% CI	P value	OR	95% CI	P value
Dulaglutide use	1.4	0.5–3.5	0.47	0.9	0.3–2.4	0.80
Age, per 10 yr increment	0.9	0.6–1.3	0.55			
Male	2.2	0.8–5.9	0.10			
HTN	1.3	0.4–4.1	0.67			
CKD	0.6	0.2–1.5	0.24			
Dyslipidemia	0.9	0.4–2.8	0.98			
HbA1c, per 1% increment	1.5	1.1–1.9	<.01	1.5	1.1–2.0	<.01
Albumin, per 1 g/dL increment	0.7	0.3–1.6	0.34			
ALT, per 30 U/L increment	0.9	0.5–1.8	0.79			
LDL, per 100 mg/dL increment	0.9	0.6–1.7	0.99			
TRIG, per 100 mg/dL increment	0.9	0.9–1.1	0.67			
SGLT2 inhibitors use	1.5	0.6–3.9	0.37			
ARB use	2.4	0.7–6.4	0.09			
Spironolactone use	1.6	0.5–4.9	0.41			
Biguanides use	2.4	0.9–6.2	0.07			
Loop diuretics use	1.1	0.3–4.2	0.89			
Thiazide use	0.4	0.1–3.7	0.45			
Statin use	1.8	0.6–5.8	0.30			

OR, odds ratio; CI, confidence interval; Yr, year; HTN, hypertension; CKD, chronic kidney disease; HF, heart failure; HbA1c, glycated hemoglobin; ALT, alanine aminotransferase; LDL, low density lipoprotein; TRIG, triglyceride; SGLT2, sodium-glucose cotransporter-2; ARB, angiotensin receptor blockers; UACR, urine albumin-to-creatinine ratio.

Risk analysis performed by using logistic regression model. Variables with P values of < 0.05 in univariate logistic regression models were included into the multivariate logistic regression model.

Supplementary Table S8. Subgroup Analysis of the Risk of UACR Increase in Patients without HF.

Character	Univariate			Multivariate		
	OR	95% CI	P value	OR	95% CI	P value
Dulaglutide use	2.8	2.0–3.9	<.01	1.7	1.2–2.6	<.01
Age, per 10 yr increment	1.4	1.3–1.6	<.01	1.2	1.1–1.4	<.01
Male	1.3	0.9–1.9	0.07			
HTN	2.0	1.4–2.9	<.01	1.4	0.9–2.2	0.18
CKD	0.7	0.5–1.1	0.09			
Dyslipidemia	1.5	1.1–2.3	0.02	1.1	0.7–1.6	0.75
HbA1c, per 1% increment	1.3	1.2–1.4	<.01	1.2	1.1–1.3	<.01
Albumin, per 1 g/dL increment	0.9	0.7–1.4	0.85			
ALT, per 30 U/L increment	0.8	0.6–0.9	0.04	0.8	0.6–1.1	0.16
LDL, per 100 mg/dL increment	0.4	0.2–0.7	<.01	0.6	0.3–1.1	0.08
TRIG, per 100 mg/dL increment	1.0	0.9–1.1	0.29			
SGLT2 inhibitors use	2.7	1.9–3.8	<.01	1.7	1.2–2.5	<.01
ARB use	1.7	1.2–2.4	<.01	1.0	0.7–1.6	0.84
Spironolactone use	1.4	0.5–3.8	0.57			
Biguanides use	3.2	2.2–4.7	<.01	1.8	1.1–2.8	0.01
Loop diuretics use	2.5	1.1–5.4	0.02	2.1	0.8–5.0	0.08
Thiazide use	1.4	0.8–2.3	0.21			
Statin use	2.3	1.5–3.4	<.01	1.3	0.8–2.0	0.33

OR, odds ratio; CI, confidence interval; Yr, year; HTN, hypertension; CKD, chronic kidney disease; HF, heart failure; HbA1c, glycated hemoglobin; ALT, alanine aminotransferase; LDL, low density lipoprotein; TRIG, triglyceride; SGLT2, sodium-glucose cotransporter-2; ARB, angiotensin receptor blockers; UACR, urine albumin-to-creatinine ratio.

Risk analysis performed by using logistic regression model. Variables with P values of < 0.05 in univariate logistic regression models were included into the multivariate logistic regression model.

Supplementary Table S9. Subgroup Analysis of the Risk of UACR Increase in Patients Using SGLT2 inhibitors

Character	Univariate			Multivariate		
	OR	95% CI	P value	OR	95% CI	P value
Dulaglutide use	1.6	0.9–2.9	0.07	1.5	0.8–2.6	0.20
Age, per 10 yr increment	1.1	0.8–1.3	0.52			
Male	1.5	0.8–2.7	0.20			
HTN	1.1	0.6–2.2	0.75			
CKD	1.4	0.8–2.5	0.24			
HF	0.5	0.2–1.0	0.06	0.5	0.2-1.1	0.10
Dyslipidemia	2.0	0.9–4.3	0.05	1.9	0.9–4.0	0.09
HbA1c, per 1% increment	1.2	0.9–1.4	0.06	1.1	0.9–1.4	0.18
Albumin, per 1 g/dL increment	1.2	0.6–2.3	0.66			
ALT, per 30 U/L increment	0.8	0.5–1.1	0.16			
LDL, per 100 mg/dL increment	0.9	0.3–2.5	0.87			
TRIG, per 100 mg/dL increment	1.0	0.9–1.1	0.71			
ARB use	0.8	0.5–1.5	0.57			
Spironolactone use	0.6	0.1–2.1	0.37			
Biguanides use	1.5	0.7–3.2	0.35			
Loop diuretics use	1.1	0.3–3.3	0.92			
Thiazide use	1.5	0.6–3.7	0.34			
Statin use	0.9	0.4–2.0	0.79			

OR, odds ratio; CI, confidence interval; Yr, year; HTN, hypertension; CKD, chronic kidney disease; HF, heart failure; HbA1c, glycated hemoglobin; ALT, alanine aminotransferase; LDL, low density lipoprotein; TRIG, triglyceride; SGLT2, sodium-glucose cotransporter-2; ARB, angiotensin receptor blockers; UACR, urine albumin-to-creatinine ratio.

Risk analysis performed by using logistic regression model. Variables with P values of < 0.06 in univariate logistic regression models were included into the multivariate logistic regression model.

Supplementary Table S10. Subgroup Analysis of the Risk of UACR Increase in Patients Not Using SGLT2 inhibitors

Character	Univariate			Multivariate		
	OR	95% CI	P value	OR	95% CI	P value
Dulaglutide use	2.6	1.8–3.9	<.01	1.7	1.1–2.6	0.01
Age, per 10 yr increment	1.4	1.2–1.6	<.01	1.3	1.1–1.5	<.01
Male	1.2	0.8–1.6	0.28			
HTN	2.1	1.4–3.4	<.01	1.5	0.8–2.4	0.15
CKD	0.6	0.4–0.8	<.01	0.6	0.4–0.9	0.01
HF	0.8	0.4–1.5	0.51			
Dyslipidemia	1.2	0.8–1.8	0.33			
HbA1c, per 1% increment	1.3	1.2–1.5	<.01	1.2	1.1–1.4	<.01
Albumin, per 1 g/dL increment	0.9	0.6–1.4	0.62			
ALT, per 30 U/L increment	0.8	0.6–1.1	0.12			
LDL, per 100 mg/dL increment	0.5	0.2–0.8	0.01	0.6	0.3–1.1	0.11
TRIG, per 100 mg/dL increment	1.0	0.9–1.1	0.66			
ARB use	2.1	1.4–3.1	<.01	1.3	0.8–2.4	0.24
Spironolactone use	1.7	0.7–24.1	0.23			
Biguanides use	3.1	2.1–4.7	<.01	1.4	0.8–2.4	0.16
Loop diuretics use	2.1	0.9–4.7	0.06			
Thiazide use	1.2	0.6–2.1	0.70			
Statin use	2.4	1.5–3.7	<.01	1.5	0.9–2.6	0.09

OR, odds ratio; CI, confidence interval; Yr, year; HTN, hypertension; CKD, chronic kidney disease; HF, heart failure; HbA1c, glycated hemoglobin; ALT, alanine aminotransferase; LDL, low density lipoprotein; TRIG, triglyceride; SGLT2, sodium-glucose cotransporter-2; ARB, angiotensin receptor blockers; UACR, urine albumin-to-creatinine ratio.

Risk analysis performed by using logistic regression model. Variables with P values of < 0.05 in univariate logistic regression models were included into the multivariate logistic regression model.

Supplementary Table S11. Subgroup Analysis of the Risk of UACR Increase in Patients Using ARBs.

Character	Univariate			Multivariate		
	OR	95% CI	P value	OR	95% CI	P value
Dulaglutide use	2.8	1.9–4.1	<.01	2.2	1.4–3.4	<.01
Age, per 10 yr increment	1.3	1.1–1.5	<.01	1.2	0.9–1.4	0.05
Male	1.0	0.7–1.4	0.83			
HTN	0.9	0.5–1.5	0.63			
CKD	0.9	0.7–1.4	0.92			
HF	0.8	0.4–1.4	0.45			
Dyslipidemia	1.1	0.7–1.8	0.59			
HbA1c, per 1% increment	1.2	1.1–1.3	<.01	1.1	0.9–1.3	0.06
Albumin, per 1 g/dL increment	0.9	0.6–1.4	0.71			
ALT, per 30 U/L increment	0.7	0.5–1.0	0.05	0.7	0.5–1.1	0.33
LDL, per 100 mg/dL increment	0.7	0.4–1.3	0.26			
TRIG, per 100 mg/dL increment	1.0	0.9–1.1	0.59			
SGLT2 inhibitors use	1.7	1.1–2.5	0.01	1.3	0.9–2.0	0.19
Spironolactone use	1.2	0.6–2.8	0.59			
Biguanides use	2.3	1.4–3.7	<.01	1.6	0.9–2.7	0.08
Loop diuretics use	1.2	0.6–2.8	0.59			
Thiazide use	1.0	0.6–1.7	0.89			
Statin use	1.5	0.8–2.6	0.16			

OR, odds ratio; CI, confidence interval; Yr, year; HTN, hypertension; CKD, chronic kidney disease; HF, heart failure; HbA1c, glycated hemoglobin; ALT, alanine aminotransferase; LDL, low density lipoprotein; TRIG, triglyceride; SGLT2, sodium-glucose cotransporter-2; ARB, angiotensin receptor blockers; UACR, urine albumin-to-creatinine ratio.

Risk analysis performed by using logistic regression model. Variables with P values of < 0.05 in univariate logistic regression models were included into the multivariate logistic regression model.

Supplementary Table S12. Subgroup Analysis of the Risk of UACR Increase in Patients Not Using ARBs.

Character	Univariate			Multivariate		
	OR	95% CI	P value	OR	95% CI	P value
Dulaglutide use	2.1	1.3–3.6	<.01	0.9	0.5–1.8	0.88
Age, per 10 yr increment	1.4	1.2–1.6	<.01	1.2	0.9–1.5	0.07
Male	2.5	1.5–4.3	<.01	1.8	0.9–3.3	0.05
HTN	2.5	1.4–4.2	<.01	2.6	1.4–4.9	<.01
CKD	0.4	0.3–0.7	<.01	0.5	0.2–0.8	0.01
HF	0.6	0.2–1.4	0.24			
Dyslipidemia	1.8	0.9–3.1	0.05			
HbA1c, per 1% increment	1.4	1.3–1.6	<.01	1.4	1.2–1.7	<.01
Albumin, per 1 g/dL increment	0.9	0.6–1.7	0.86			
ALT, per 30 U/L increment	0.9	0.6–1.2	0.39			
LDL, per 100 mg/dL increment	0.3	0.1–0.8	0.01	0.6	0.2–1.7	0.33
TRIG, per 100 mg/dL increment	1.0	0.9–1.2	0.43			
SGLT2 inhibitors use	4.1	2.3–7.3	<.01	2.8	1.5–5.5	<.01
Spironolactone use	0.6	0.1–5.1	0.66			
Biguanides use	3.7	2.1–6.3	<.01	1.5	0.7–3.2	0.24
Loop diuretics use	3.4	1.1–10.6	0.03	4.4	1.2–15.4	0.02
Thiazide use	1.4	0.6–3.1	0.98			
Statin use	2.3	1.4–4.1	<.01	1.2	0.6–2.3	0.58

OR, odds ratio; CI, confidence interval; Yr, year; HTN, hypertension; CKD, chronic kidney disease; HF, heart failure; HbA1c, glycated hemoglobin; ALT, alanine aminotransferase; LDL, low density lipoprotein; TRIG, triglyceride; SGLT2, sodium-glucose cotransporter-2; ARB, angiotensin receptor blockers; UACR, urine albumin-to-creatinine ratio.

Risk analysis performed by using logistic regression model. Variables with P values of < 0.05 in univariate logistic regression models were included into the multivariate logistic regression model.

Supplementary Table S13. Subgroup Analysis of the Risk of UACR Increase in Patients Using Biguanides.

Character	Univariate			Multivariate		
	OR	95% CI	P value	OR	95% CI	P value
Dulaglutide use	2.3	1.6–3.3	<.01	2.1	1.4–3.0	<.01
Age, per 10 yr increment	1.2	1.1–1.3	0.02	1.1	0.9–1.3	0.10
Male	1.2	0.8–1,8	0.27			
HTN	1.3	0.9–2.0	0.21			
CKD	1.1	0.8–1.6	0.56			
HF	0.7	0.4–1.3	0.31			
Dyslipidemia	0.9	0.6–1.5	0.86			
HbA1c, per 1% increment	1.1	0.9–1.2	0.10			
Albumin, per 1 g/dL increment	0.9	0.6–1.4	0.69			
ALT, per 30 U/L increment	0.8	0.6–1.1	0.22			
LDL, per 100 mg/dL increment	0.7	0.4–1.3	0.30			
TRIG, per 100 mg/dL increment	1.0	0.9–1.1	0.56			
SGLT2 inhibitors use	1.6	1.1–2.4	<.01	1.5	1.1–2.2	0.04
ARB use	1.2	0.8–1.8	0.32			
Spironolactone use	0.9	0.4–2.1	0.83			
Loop diuretics use	1.3	0.5–3.5	0.63			
Thiazide use	0.9	0.6–1.7	0.94			
Statin use	1.2	0.7–2,1	0.41			

OR, odds ratio; CI, confidence interval; Yr, year; HTN, hypertension; CKD, chronic kidney disease; HF, heart failure; HbA1c, glycated hemoglobin; ALT, alanine aminotransferase; LDL, low density lipoprotein; TRIG, triglyceride; SGLT2, sodium-glucose cotransporter-2; ARB, angiotensin receptor blockers; UACR, urine albumin-to-creatinine ratio.

Risk analysis performed by using logistic regression model. Variables with P values of < 0.05 in univariate logistic regression models were included into the multivariate logistic regression model.

Supplementary Table S14. Subgroup Analysis of the Risk of UACR Increase in Patients Not Using Biguanides.

Character	Univariate			Multivariate		
	OR	95% CI	P value	OR	95% CI	P value
Dulaglutide use	1.6	0.8–3.5	0.21	0.5	0.2–1.4	0.19
Age, per 10 yr increment	1.3	1.1–1.8	0.04	1.3	1.1–1.8	0.02
Male	1.5	0.8–2.8	0.19			
HTN	7.8	2.4–25.7	<.01	4.0	1.1–14.3	0.03
CKD	0.6	0.3–1.1	0.10			
HF	0.9	0.4–2.3	0.99			
Dyslipidemia	2.0	1.1–4.2	0.04	1.4	0.6–3.3	0.43
HbA1c, per 1% increment	1.6	1.3–1.8	<.01	1.6	1.3–1.9	<.01
Albumin, per 1 g/dL increment	1.3	0.6–2.5	0.50			
ALT, per 30 U/L increment	0.6	0.3–1.1	0.10			
LDL, per 100 mg/dL increment	0.4	0.2–1,2	0.09			
TRIG, per 100 mg/dL increment	1.0	0.9–1.2	0.76			
SGLT2 inhibitors use	3.6	1.6–8.0	<.01	2.5	1.0–1.9	0.05
ARB use	2.0	1.1–3.7	0.03	0.9	0.4–1.9	0.74
Spironolactone use	2.3	0.5–11.4	0.31			
Loop diuretics use	4.3	1.7–10.5	<.01	2.2	0.8–6.1	0.12
Thiazide use	1.5	0.4–5.4	0.53			
Statin use	2.0	1.1–3.8	0.03	1.5	0.7–3.4	0.32

OR, odds ratio; CI, confidence interval; Yr, year; HTN, hypertension; CKD, chronic kidney disease; HF, heart failure; HbA1c, glycated hemoglobin; ALT, alanine aminotransferase; LDL, low density lipoprotein; TRIG, triglyceride; SGLT2, sodium-glucose cotransporter-2; ARB, angiotensin receptor blockers; UACR, urine albumin-to-creatinine ratio.

Risk analysis performed by using logistic regression model. Variables with P values of < 0.05 in univariate logistic regression models were included into the multivariate logistic regression model.

Supplementary Table S15. Subgroup Analysis of the Risk of UACR Increase in Patients Using Statins.

Character	Univariate			Multivariate		
	OR	95% CI	P value	OR	95% CI	P value
Dulaglutide use	2.3	1.6–3.3	<.01	1.5	1.1–2.2	0.03
Age, per 10 yr increment	1.3	1.2–1.5	<.01	1.3	1.1–1.5	<.01
Male	1.4	0.9–1.9	0.08			
HTN	1.6	1.1–2.4	0.02	1.4	0.9–2.2	0.11
CKD	0.9	0.6–1.3	0.56			
HF	0.7	0.4–1.2	0.18			
Dyslipidemia	1.3	0.8–2.1	0.23			
HbA1c, per 1% increment	1.2	1.1–1.4	<.01	1.2	1.1–1.3	0.01
Albumin, per 1 g/dL increment	1.1	0.7–1.6	0.79			
ALT, per 30 U/L increment	0.8	0.6–1.1	0.05			
LDL, per 100 mg/dL increment	0.6	0.4–1.1	0.12			
TRIG, per 100 mg/dL increment	1.0	0.9–1.1	0.37			
SGLT2 inhibitors use	1.9	1.3–2.7	<.01	1.5	1.1–2.2	0.03
ARB use	1.4	0.9–2.0	0.10			
Spironolactone use	1.2	0.6–2.6	0.62			
Biguanides use	2.4	1.5–3.6	<.01	1.7	1.1–2.8	0.02
Loop diuretics use	1.9	0.9–3.9	0.10			
Thiazide use	0.9	0.6–1.7	0.93			

OR, odds ratio; CI, confidence interval; Yr, year; HTN, hypertension; CKD, chronic kidney disease; HF, heart failure; HbA1c, glycated hemoglobin; ALT, alanine aminotransferase; LDL, low density lipoprotein; TRIG, triglyceride; SGLT2, sodium-glucose cotransporter-2; ARB, angiotensin receptor blockers; UACR, urine albumin-to-creatinine ratio.

Risk analysis performed by using logistic regression model. Variables with P values of < 0.05 in univariate logistic regression models were included into the multivariate logistic regression model.

Supplementary Table S16. Subgroup Analysis of the Risk of UACR Increase in Patients Not Using Statins.

Character	Univariate			Multivariate		
	OR	95% CI	P value	OR	95% CI	P value
Dulaglutide use		1.5–56.5	<.01	1.6	0.7–3.6	0.22
Age, per 10 yr increment	1.3	1.1–1.8	0.04	1.1	0.8–1.4	0.53
Male	1.1	0.6–2.2	0.71			
HTN	2.2	1.0–4.9	0.04	2.0	0.7–5.5	0.17
CKD	0.4	0.2–0.7	<.01	0.4	0.2–0.9	0.02
HF	0.9	0.3–2.6	0.79			
Dyslipidemia	0.9	0.4–1.7	0.65			
HbA1c, per 1% increment	1.5	1.3–1.8	<.01	1.3	1.1–1.6	<.01
Albumin, per 1 g/dL increment	0.6	0.3–1.3	0.17			
ALT, per 30 U/L increment	0.9	0.6–1.3	0.56			
LDL, per 100 mg/dL increment	0.4	0.1–1.2	0.08			
TRIG, per 100 mg/dL increment	1.0	0.9–1.2	0.73			
SGLT2 inhibitors use	5.0	2.1–11.6	<.01	3.4	1.3–8.8	0.01
ARB use	2.1	1.1–4.3	0.03	0.9	0.4–2.3	0.96
Spironolactone use	0.5	0.1–4.1	0.53			
Biguanides use	3.8	1.9–7.6	<.01	1.4	0.6–3.3	0.44
Loop diuretics use	1.5	0.3–7.2	0.61			
Thiazide use	3.2	0.9–10.8	0.06			

OR, odds ratio; CI, confidence interval; Yr, year; HTN, hypertension; CKD, chronic kidney disease; HF, heart failure; HbA1c, glycated hemoglobin; ALT, alanine aminotransferase; LDL, low density lipoprotein; TRIG, triglyceride; SGLT2, sodium-glucose cotransporter-2; ARB, angiotensin receptor blockers; UACR, urine albumin-to-creatinine ratio.

Risk analysis performed by using logistic regression model. Variables with P values of < 0.05 in univariate logistic regression models were included into the multivariate logistic regression model.
